# Geospatial clustering, seasonal trend and forecasting of Kyasanur Forest Disease in the state of Goa, India, 2015–2018

**DOI:** 10.1186/s41182-020-00213-y

**Published:** 2020-04-28

**Authors:** Annet Oliveira, Kalaiselvi Selvaraj, Jaya Prasad Tripathy, Utkarsh Betodkar, Jagadish Cacodcar, Nikhita Quadros, Abhijit Wadkar

**Affiliations:** 1 Directorate of Health Services, Panaji, Goa India; 2 Inegrated Disease Surveillence Programme, Panaji, Goa India; 3 Department of Community Medicine, All India Institute of Medical Sciences, Nagpur, India; 4 Department of Preventive and Social Medicine, Goa Medical College, Bambolim, Goa India

**Keywords:** Tropical fever, Hemorrhagic fever, Tick-borne fever, Kyasanur Forest Disease, Disease surveillance, Seasonal trend, Count model, Time series analysis, Spatio-temporal distribution

## Abstract

**Introduction:**

Five states in India are reporting sporadic outbreaks of Kyasanur Forest Disease (KFD). Goa experienced an outbreak of KFD in 2015. It remains as an important differential diagnosis for tropical fever in the endemic regions. Few studies among neighboring two states (Karnataka and Kerala) have described the epidemiological characteristics of KFD. However, there is no study which describes the same among cases in the state of Goa. Hence, we planned to understand the epidemiology (time, place, and person distribution) of the disease including seasonal pattern with forecasting using zero-inflated negative binomial regression and time series models. We also explored geo-spatial clustering of KFD cases in Goa during 2015–2018 which would help design effective intervention to curb its transmission in Goa.

**Results:**

Blood samples of all suspected cases of KFD during 2015 to 2018 were tested using reverse transcriptase-polymerase chain reaction technique. Reports of these results were periodically shared with the state surveillance unit. Records of 448 confirmed cases of KFD available at the State Integrated Disease Surveillance Programme were analyzed. The mean (SD) age of the patients was 41.6 (14.9) years. Of 143 cases with documented travel history, 135 (94.4%) had history of travel to forest for cashew plucking. Two thirds of cases (66.3%) did not receive KFD vaccine prior to the disease. Case fatality rate of 0.9% was reported. Seasonal peaks were observed during January to April, and forecasting demonstrated a peak in cases in the subsequent year also during January–April persisting till May. Around 40 villages located along the Western Ghats had reported KFD, and affected villages continued to report cases in the subsequent years also. Case density-based geographic maps show clustering of cases around the index village.

**Conclusion:**

Most of the confirmed cases did not receive any vaccination. KFD cases in Goa followed a specific seasonal pattern, and clustering of cases occurred in selected villages located in North Goa. Most of the patients who had suffered from the disease had visited the forest for cashew plucking. Planning for public health interventions such as health education and vaccination campaigns should consider these epidemiological features.

## Introduction

Kyasanur Forest disease (KFD) is a tick-borne viral hemorrhagic disease transmitted by the infected tick bite of *Haemaphysalis spinigera*. It was first discovered in 1957 in India in the Kyasanar forest of Shimoga district, Karnataka, after reported monkey deaths. KFD was confined to five districts of Karnataka until 2012. Since then, this disease is gradually expanding to adjoining districts and states located along the lines of the Western Ghats. Currently, five states in India report sporadic outbreaks of KFD, namely Karnataka, Tamil Nadu, Kerala, Maharashtra, and Goa [1, 2].

Annually 300–400 KFD cases are reported from India in recent years with the case fatality rate ranging from 2–10%. KFD virus is a highly pathogenic virus which has a flu-like presentation. Till date, there is no specific treatment available for KFD and supportive therapy is the mainstay of management [3].

The tick species is widely distributed in the deciduous and evergreen forests of South India where the disease is endemic. Of the several stages of ticks, nymphal forms are considered to be highly anthropophilic. These nymphal forms are highly prevalent during the post-monsoon period in the months of December–May [3]. The KFD cases are seen to peak from January to March [4]. Local villagers staying in and around the forest area frequently visit the forest for collection of firewoods and grass and get infected through tick bites. The recent ecological changes such as encroachment of forest region, shifting the cultivation to forest areas, and climate change has increased the risk of transmission [5–7]. The spread of the disease to newer areas is a major cause for concern [8].

The first case of KFD in Goa was reported in March 2015 in Palli village of Sattari taluka, a border district between Goa and Karnataka. Since then, a total of 462 KFD have been reported in the State of Goa from 2015 to 2018. The blood serum samples of the clinically suspected fever cases were tested by Manipal Centre for Virus Research, Karnataka, using the reverse transcription-polymerase chain reaction (RT-PCR) method. Case investigation was also carried out to understand the origin and spread of the outbreak.

Studies from Karnataka and Kerala have reported the epidemiological characteristics of KFD cases. However, there is no study which describes the same among cases in the state of Goa except a case series reported among cashew nut workers [8–12]

We have tried to understand the geographical distribution of KFD cases and their spread on a village level map. In a resource-poor setting like India, mapping clusters of KFD cases would be helpful in the identification of geographical hot spots and planning effective strategies for control and elimination programs. These maps would provide village level information about the spatial patterns of the disease, which is crucial for public health officials to prioritize their activities at identified clusters.

We have also explored the seasonality of KFD cases in Goa as it is an important epidemiological attribute. Previous outbreaks from Karnataka and Kerala have been reported over a wide span of 8 months from November to June with indistinct peaks, with minimal information from the state of Goa [8]. Analyzing the seasonality of occurrence and forecasting the incidence of KFD cases will help in deciding the timing of interventions. This study will generate local evidence to sensitize the policymakers for sustainable support for KFD control and surveillance in Goa. The study findings will also add to the limited existing literature on the epidemiology of this disease. Thus, we planned to comprehensively investigate the epidemiology of the disease in Goa since 2015 with the following specific objectives: (i) describe the socio-demographic and clinical characteristics of confirmed KFD cases, (ii) assess the spatial distribution and clustering of KFD cases in the state of Goa, and (iii) analyze the trend and forecast KFD incidence using time series forecasting methods.

## Methods

### General setting

**Fig. 1:**
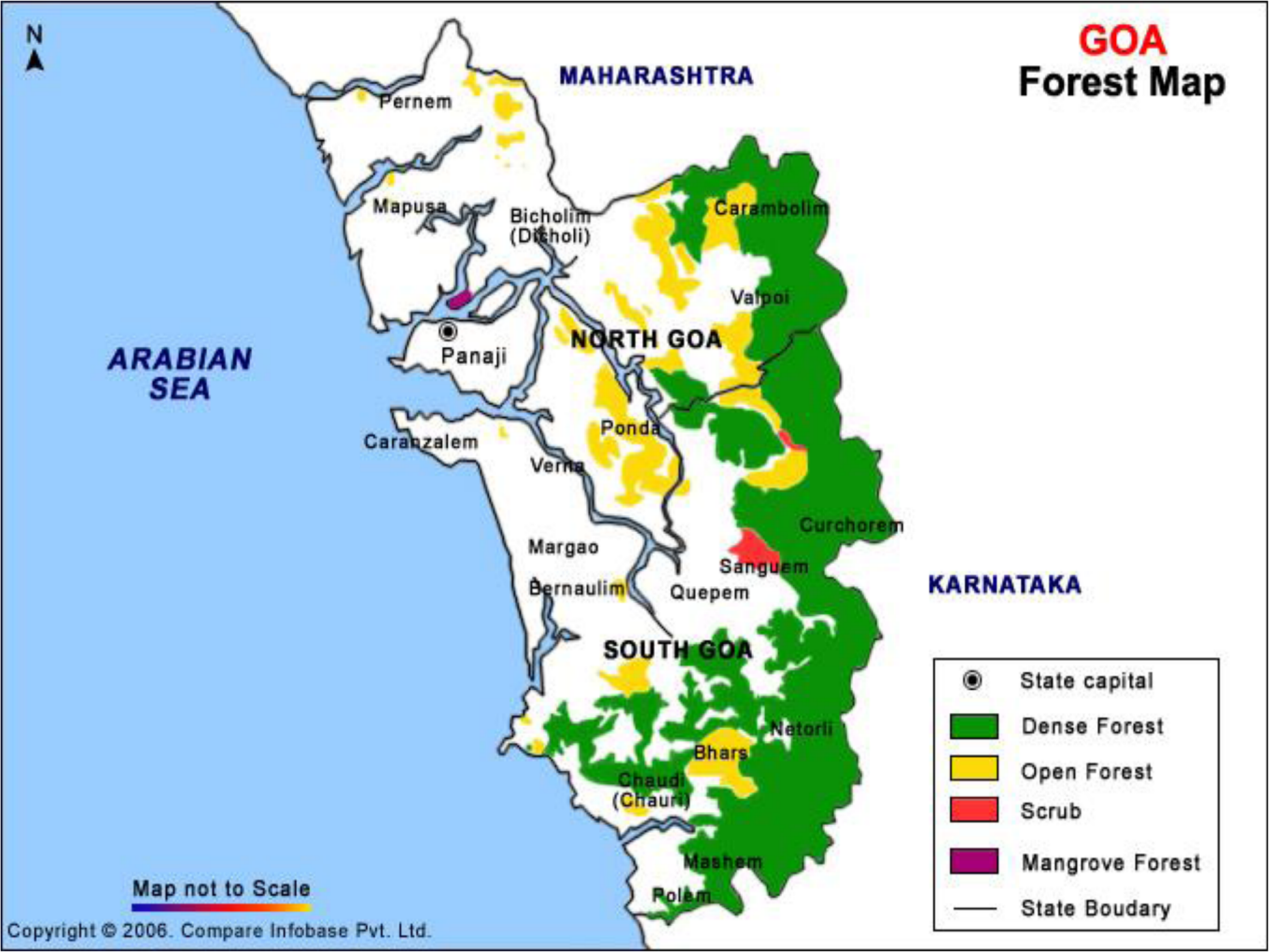
Forest map of Goa showing the distribution and type of forests

Goa is a state located along the coastal region of Western India with a total of 402 villages. The border areas of Goa have many dense forest regions which are endemic for KFD due to the presence of ticks in those regions. This region is also considered to be one of the internationally recognized bio-diversity hot spots. A map of Goa showing the forest region is displayed in Fig. 1.

### Specific setting

In Goa, the KFD outbreak was first reported in Pali village of Satteri in 2015; since then, there has been a spread to other villages. Following this, the Government of Goa permitted the Manipal Centre for Virus Research (MCVR), Karnataka, in conducting “hospital-based surveillance of acute febrile illness” at Community Health Centre Valpoi and District Hospital, North Goa. Due to lack of testing facility in Goa, all suspected cases of KFD were tested at the MCVR using the RT-PCR technique. A suspected case of KFD is defined as a patient of any age presenting with acute onset of high-grade fever with any of the following: headache/myalgia/ prostration/extreme weakness/nausea/vomiting/diarrhea/occasionally neurological/hemorrhagic manifestation AND/OR [3]

- Rule out common etiologies of acute febrile illness prevalent on the area (dengue/DHF, typhoid, malaria, etc.)
- History of exposure to tick bite
- Travel and/or living in and around forest area where recent monkey deaths have been reported

In case of any suspected case of KFD, blood sample is collected from the health facility, government or private, or by the MCVR unit directly and tested at the MCVR. The test result is then reported to the state Integrated Disease Surveillance Project (IDSP) unit.

**Fig. 2:**
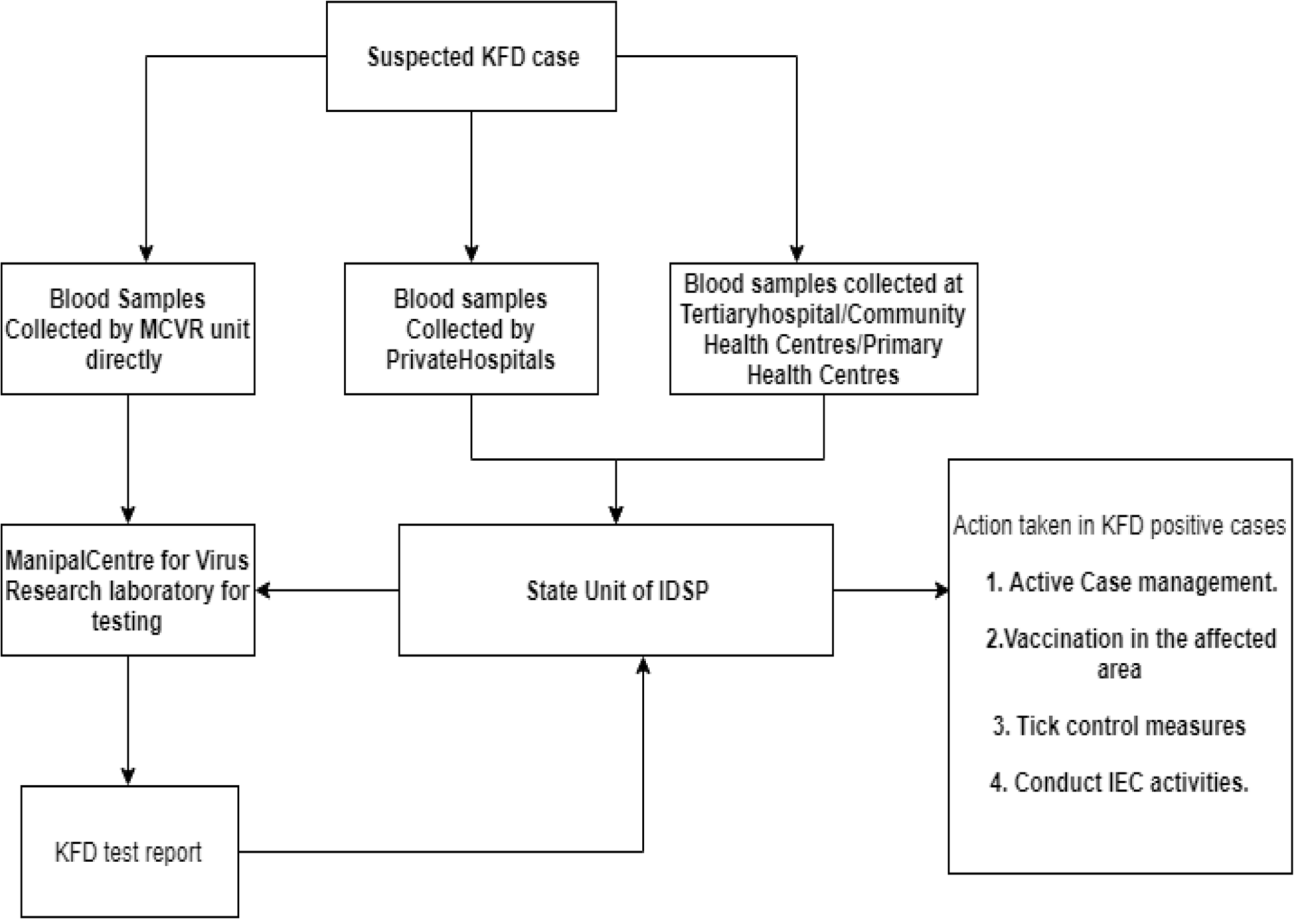
Flow chart showing diagnosis and management of suspected cases of Kyasanur Forest Disease in the state of Goa, India, during 2015–2018

The protocol for testing of suspected cases of KFD and public health response in case of confirmed case of KFD is given in Fig. 2.

### Study design

The non-spatial data were collected through the routine case investigation reports maintained at the IDSP Goa and admission records of KFD patients admitted at CHC, Valpoi, and Goa Medical College. For time series analysis, month-wise cases of KFD for the period 2015–2018 were compiled from the surveillance reports.

### Study population

A total of 448 laboratory-confirmed cases of KFD reported to the IDSP during the period 2015–2018 in Goa, India, constituted the study population.

### Data variables, sources of data, and data collection

A unique ID was given to each patient whose records were available at IDSP state unit Goa. The demographic profile, i.e., age, sex, and village, of the patients was collected from laboratory reports sent by MCVR, whereas occupation and vaccination history were obtained from the investigation report of KFD-positive cases maintained under IDSP since November 2016. All cases prior to 9 March 2016 (except for Pali village) were considered as non-vaccinated as vaccination was not given to other villages prior to that date. Also, vaccination registers were inspected to cross-check if the KFD patients had been vaccinated. The clinical profile of the patients was obtained from the case papers of admitted patients at CHC Valpoi and Goa Medical College. The admission book from CHC Valpoi was used to obtain the date of admission, date of discharge, and date of transfer to higher referral center. Spatial data included the geolocation of every village, whereas the number of cases of KFD in each village was the attribute to depict geospatial distribution and clustering of cases. A village shapefile (.*shp*) of Goa state was used for geo-mapping.

### Analysis and statistics

Data were double entered and validated using EpiData entry (version 3.1) and analyzed using Epidata analysis (V2.2.2.183). Patient demographic characteristics such as age and hospital stay were summarized in the form of mean (SD) or median (IQR). Clinical characteristics and vaccination status were presented as frequencies and percentages.

#### Geospatial distribution

Using the village administrative map of Goa, cumulative cases per 1000 population is depicted in the form of density map through Quantum Geographic Information System (QGIS) software.

#### Spatial clustering

The spatial autocorrelation was analyzed by global Moran’s I statistic. We used the first-order Queen’s contiguity rule to define spatial neighborhood relationship. Moran’s *I* range from + 1 (highly positive autocorrelation) to − 1 (highly negative autocorrelation), while 0 corresponds to spatially random distribution. It tests whether objects with similar attribute values lie close to each other [13].

The local Moran’s *I* statistic was used to identify local clusters and local spatial outliers. This allows for a classification of the significant locations as high-high and low-low *spatial clusters* and high-low and low-high *spatial outliers*. A positive value for *I* indicates that a feature has neighboring features with similarly high or low attribute values which forms clusters; a negative value for *I* indicates that a feature has neighboring features with dissimilar values which forms an outlier. A high attribute value surrounded primarily by low values is a high-low outlier, and a low value surrounded primarily by high values is a low-high outlier [14, 15]. GeoDa software version 1.14 was used for spatial autocorrelation.

#### Forecasting

Distribution of cases since 2015 January to December 2018 was approached through applied time series modeling and count data static regression modeling. Both these models were used to compare and help inform model adequacy. The model which yielded the lowest Akaike information criteria (AIC) and Bayesian information criteria (BIC) was considered for forecasting.

##### Estimation based on time series model

Month-wise distribution of confirmed cases of KFD was plotted to show the seasonal trend. Monthly data for 36 months (2015–2018) was analyzed for seasonal trend and forecasting. Seasonal trend of KFD was analyzed by Seasonal Auto-Regressive Integrated Moving Average (SARIMA) model using time-domain approach through Gretl software (gretl 1.7.9cvs) as per the following hierarchical steps/procedures:

1. Initially, time series plot was drawn to identify the pattern of occurrence of month-wise cases of KFD.
2. Correlogram was drawn to identify the appropriate number of lag periods and moving average to fit the model (p,d,q). In correlogram, number of spikes in auto-correlation function and partial auto-correlation function projecting beyond zero was used to guide moving average and auto-regression model parameters respectively.
3. To achieve stationary time series data, seasonal difference with ARIMA was estimated using Augmented Dickey-Fuller test.
4. Among the various postulated combinations of auto-regression and moving average models, the possible best fit model was estimated through exact maximum likelihood estimation, and the model which results in the lowest Akaike information criteria method was considered as the best predictive model
5. Model diagnostics were checked using the correlogram for residual plot.
6. At the end, using the fitted SARIMA model forecasting was predicted for the next 12-month period.

##### Estimation based on count model

Cases were summarized as mean and standard deviation. The number of KFD cases was predicted using count regression models by using the number of observed KFD cases as dependent variables and time period as the independent variable. Since the conventional Poisson count model assumes that mean is equal to variance, data dispersion was checked to see whether this assumption was violated. Overdispersion of data was assessed using likelihood ratio test of alpha. The likelihood ratio test parameter alpha = 5.7 was significantly different from zero thereby indicating overdispersion of data. So the choice of model was more in favor of negative binomial model compared to Poisson. Also, out of 48 months observations, 25 months did not show any cases during that month. Hence, to circumvent this, zero-inflated negative binomial modeling (ZINB) was applied as the final model.

Among the time series and count models, the zero-inflated negative binomial model gave the precise coefficient parameter and the lowest AIC and BIC (Bayesian information criteria) parameter.

## Results

Among the 448 confirmed KFD patients, the mean (SD) age of the patients was 41.6 (14.9) years; less than half were males 212 (47.3%). Information about hospital admission and stay was available in 382 cases, of whom 381 were treated in inpatient settings who stayed in the hospital for median (IQR) duration of 5 (4–6) days. Nearly three fourths (*n* = 282, 73.8%) of the patients were treated in a CHC and 86 (22.5%) got treated in medical college. About two-thirds (297, 66.3%) were unvaccinated, and another 105 (23.4%) had no information on vaccination status (Table 1).

**Table 1:** Demographic and clinical characteristics of patients diagnosed with Kyasanur Forest Disease during 2015–2018 in Goa, India

Characteristics	Categories	*N* = 448 (%)
**Age in years, (presented as mean (SD))**		41.6 (14.9)
**Age group (in years)**	0–14	16 (3.6)
	15–44	232 (51.8)
	44–64	167 (37.3)
	65 and above	33 (7.4)
**Sex**	Male	212 (47.3)
	Female	236 (52.7)
**Occupation**	Home maker	87 (40.3)
	Farming	9 (4.2)
	Working in cashew plantation/plucking	32 (14.8)
	Laborer	26 (12)
	Student	25 (11.6)
	Others	37 (17.1)
**Place of Admission**	Community health center	282 (73.8)
	Medical college	86 (22.5)
	Private sector	1 (0.3)
	District hospital	9 (2.4)
	Primary health center	3 (0.8)
**Duration between onset and hospital admission (days)**	Median (IQR)	4 (2–5)
**Duration of hospital stay (days)**	Median (IQR)	5 (4–6)
**Deaths**	No	444 (99.1)
	Yes	4 (0.9)
**Vaccination status**	Not vaccinated	297 (66.3)
	One dose	37 (8.3)
	Two doses	4 (0.9)
	Three doses	5 (1.1)
	Not documented	105 (23.4)
**History of travel to forest**	Yes	135 (30.1)
	No	08 (1.8)
	Not documented	305 (68.1)

*SD* standard deviation, *IQR* inter-quartile range

History of travel to forest was obtained among 143 patients through case records, of whom 135 (94.4%) patients had traveled to the forest during the cashew plucking season.

### Seasonal trend: time series analysis

**Fig. 3:**
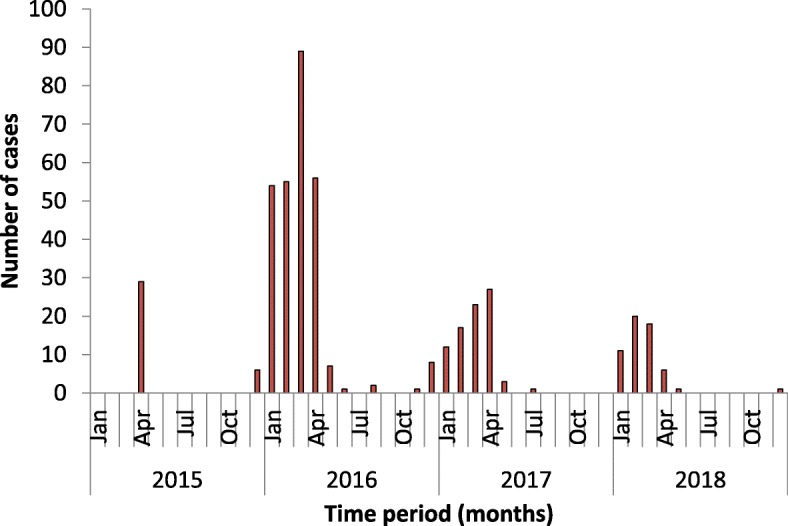
Bar chart showing the seasonality of Kyasanur Forest Disease cases in the state of Goa, India, during 2015–2018

**Fig. 4:**
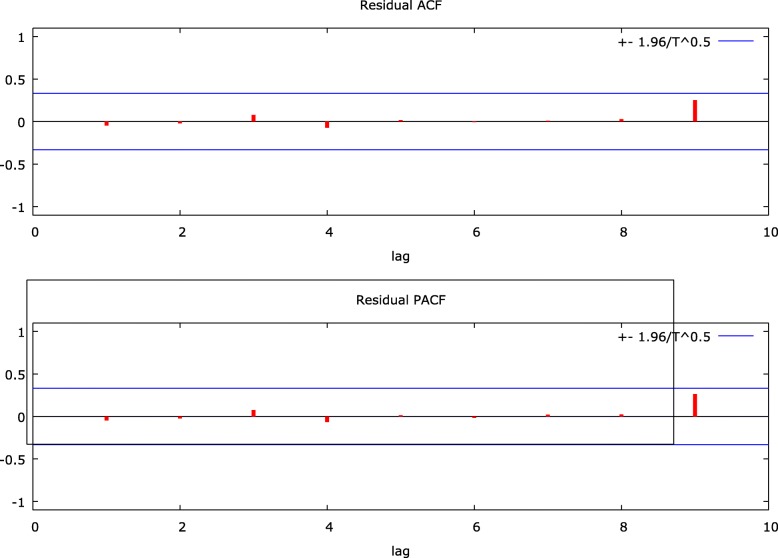
Correlogram of the original time series of cases of KFD in Goa, India, during 2015–2018

The time series plot depicts a clear seasonality during the first quarter of the year (January–April) (Fig. 3). The correlogram plots demonstrated the need to include first-order auto-regression (AR) and first-order moving average (MA) to build the model (Fig. 4).

**Fig. 5:**
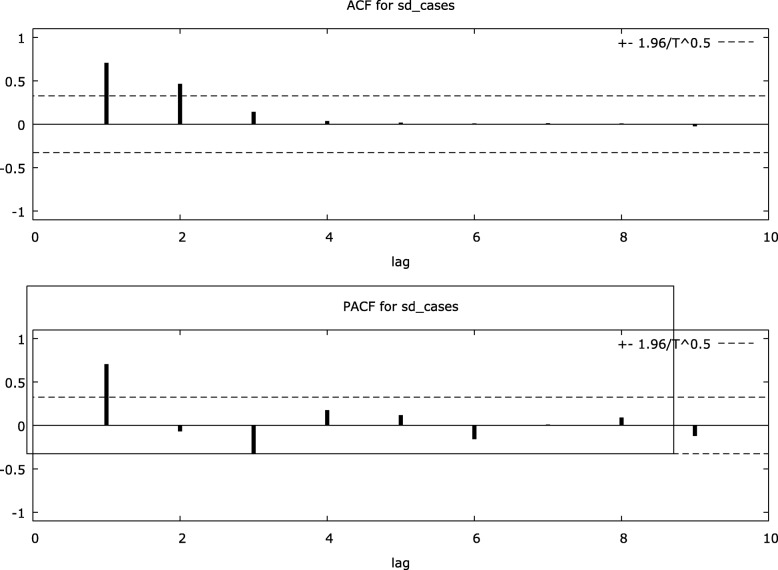
Correlogram of the seasonally differenced time series of cases of Kyasanur Forest Disease in Goa, India, during 2015–2018

Seasonal difference using augmented Dickey-Fuller test shows significant trend in seasonality (*p* = 0.04). Similarly, correlogram for seasonal differenced cases had shown first-order AR and second-order MA to be included in the model (Fig. 5).

Various combinations of first- or second-order AR with first- and second-order MA and seasonal ARIMA models were estimated using the exact maximum likelihood method. Details of AIC and BIC obtained in each model is given in (Table 2). SARIMA (1,1,1) (1,1,2)_12_ had resulted in the lowest AIC and BIC values.

**Table 2:** Comparison of Akaike information criteria (AIC) value of SARIMA models to find the best fit model

SARIMA (p,d,q) (P,D,Q)_**12**_ model	AIC value	BIC value
(2,0,2) (0,0,0)	390.7	410.97
(2,1,2) (0,0,0)_12_	391.6	402.69
(2,1,2) (1,0,1)_12_	392.36	407.16
(2,1,2) (1,1,1)_12_	301.9	314.34
(1,1,2) (1,1,1)_12_	300.4	311.28
(2,1,1) (1,1,1)_12_	300.38	311.27
**(1,1,1) (1,1,2)**_**12**_	**300.09**	**310.99**

*SARIMA* Seasonal Auto-Regressive Integrated Moving Average model

(p,d,q) (P,D,Q)_12_; *p* auto-regression function, *d* difference, *q* moving average, *P* seasonal auto-regression function, *D* seasonal difference, *Q* seasonal moving average

**Fig. 6:**
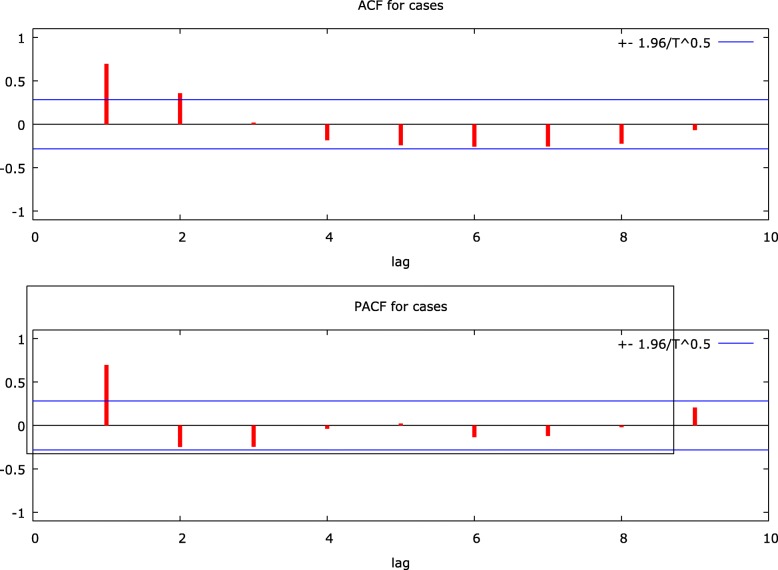
Correlogram of the residual series of cases of Kyasanur Forest Disease in Goa, India, during 2015–2018

**Fig. 7:**
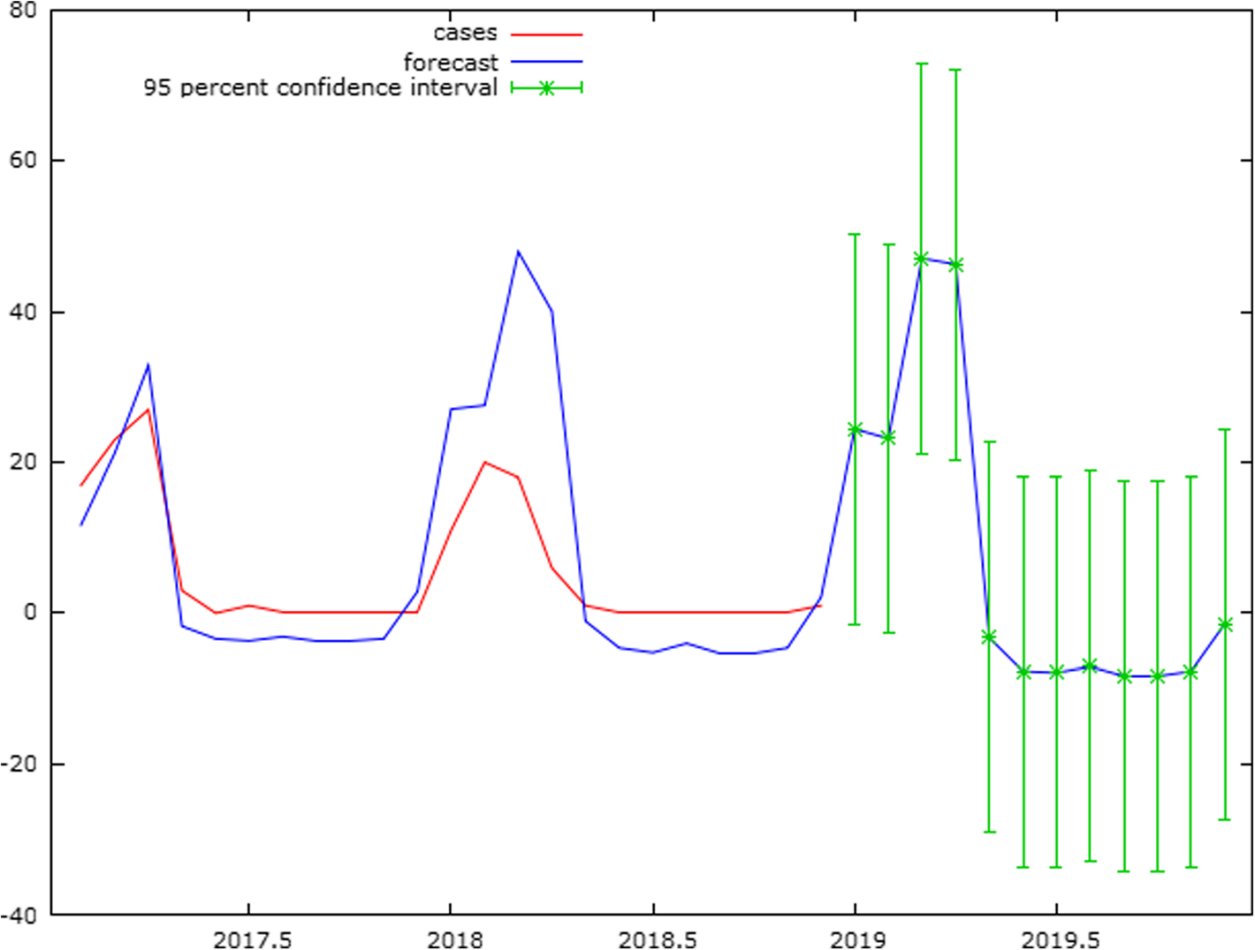
Actual and fitted cases of Kyasanur Forest Disease in Goa, India, showing a seasonal trend using SARIMA model

Also, the correlogram for residual plot of the abovementioned model also did not show any significant variance in spikes (Fig. 6). Hence, the SARIMA model (1,1,1) (1,1,2)_12_ was considered as the best fit model to forecast cases of KFD during 2019. The forecasting in 2019 shows that cases will peak during January to April and continue till May with nil reporting during the remaining months (Fig. 7).

Estimated model parameters for various count models are given in Table 3.

**Table 3:** Model parameters including the model coefficient and its confidence intervals for various count models used in prediction of KFD cases in Goa 2015–2018

Model	Co-efficient	Log-likelihood	AIC	BIC
Poisson	− 0.027 (− 0.03 to − 0.02)	− 607.90	1219.74	223.48
Negative log binomial	− 0.048 (− 0.11 to 0.016)	− 120.36	246.71	252.32
Zero-inflated negative binomial	− 0.057 (− 0.10 to − 0.02)	− 88.04	186.10	195.40

**Fig. 8:**
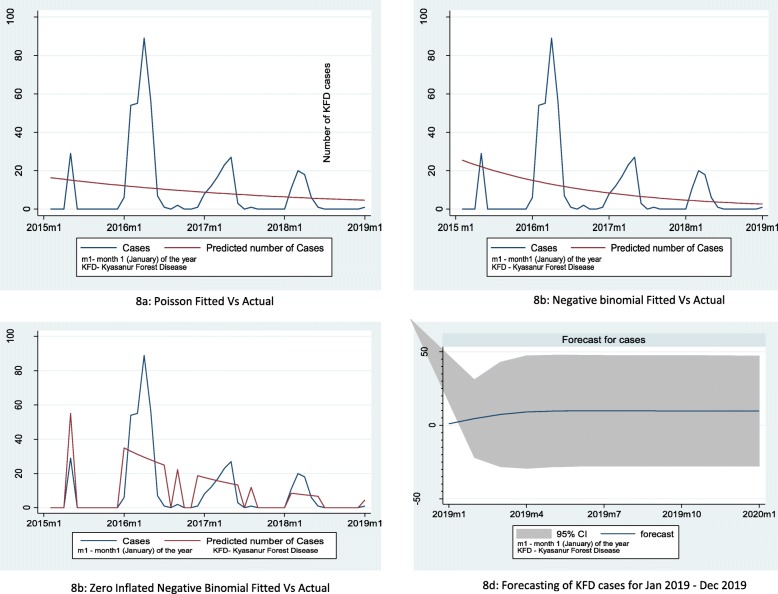
Actual and fitted cases of Kyasanur Forest Disease in Goa, India, using various Poisson regression models

The best count model (ZINB) had the lowest AIC and BIC values of 186.1 and 195.4, whereas the best time series model SARIMA model (1,1,1) (1,1,2)_12_ had the lowest AIC value of 300. Similar to time series model, the ZINB regression model also showed the cases will peak during the first 4 months of the year, and it also predicted in the future upcoming year, the cases will follow an inclining trend during the initial 4 months; thereafter, it will be plateau (Table 3 and Fig. 8).

### Pattern of geographical spread

**Fig. 9:**
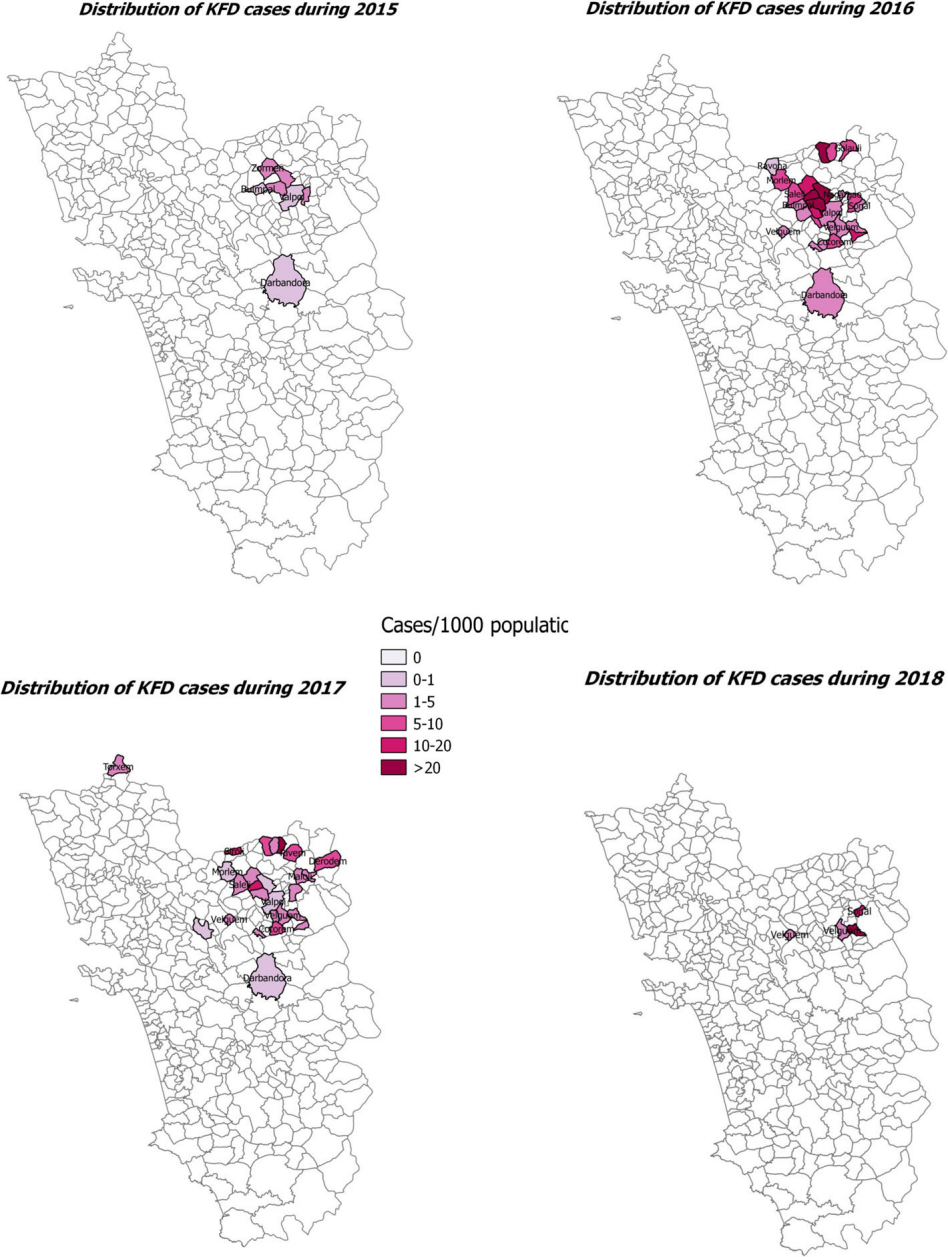
Spatio-temporal distribution of cases of Kyanasur Forest Disease in Goa, India, during 2015–2018

A total of 40 villages were affected due to KFD outbreak. In 2015, eight villages got affected, and except one, all villages continued to report KFD cases in the subsequent 2 years. The year 2016 had the maximum number of villages (31) affected due to KFD. Of these 31 villages, 18 villages continued to have the outbreak of KFD in the next year also. Of the 27 villages reported KFD in 2017, 19 had reported KFD previously. Though the number of villages affected was least in 2018, the case rate was more compared to the year 2015 (in 2015, the average case rate was 3.8 per 1000, whereas in the year 2018, the average case rate was 18.9 per 1000 population, *p* < 0.0001). About 65% of all cases have been reported from seven villages: Querim (13%), Mauzi (11.3%), Compordem (8.2%), Choraundem (8.1%), Dabem (7.4%), Pale (6.4%), and Carambolim-Buzruco (5.8%) (Fig. 9).

#### Spatial clustering analysis

**Fig. 10:**
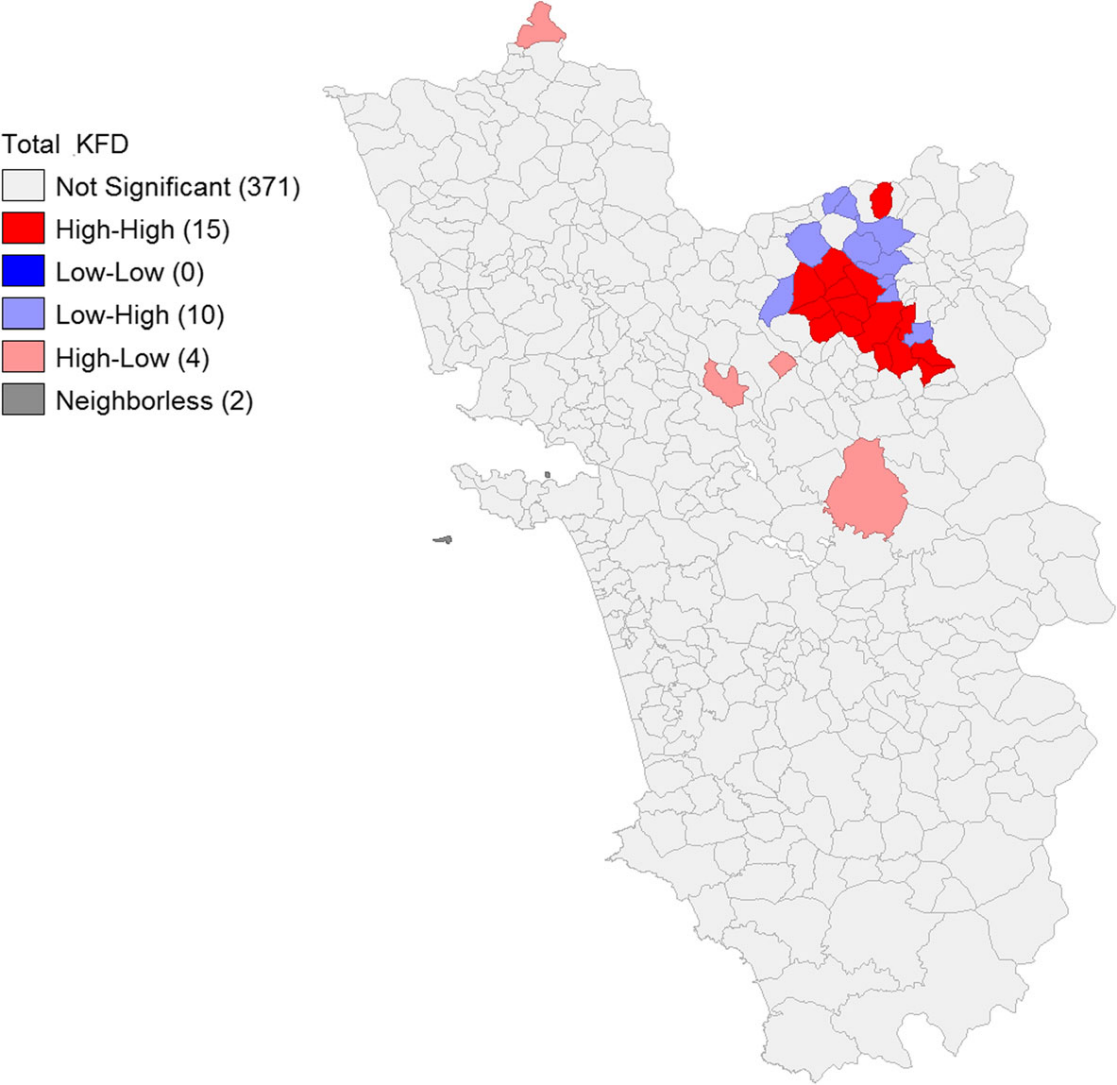
Hot spots and cold spots of cases of Kyasanur Forest Disease in Goa, 2015–2018

The Moran’s *I* statistic shows significant spatial clustering (0.314, *p* value = 0.001). Hot spot analysis revealed that local clustering of KFD cases varied spatially across the state of Goa. The spread of statistically significant hot spots (red color features) can be visualized in Fig. 10 showing a contiguous pattern. The major KFD hot spots (high-high) were located in 15 villages, high-low outliers in 4, and low-high outliers in 10 villages.

## Discussion

This study is based on the surveillance data from IDSP Goa reporting outbreak of KFD in 40 affected villages during the period 2015–2018. We describe here the epidemiology of KFD in terms of time (seasonality of occurrence), place (geo-spatial distribution), and person distribution. The study had some interesting findings which are discussed below.

First, about 90% of the cases belonged to the age group 15–64 years. Similar studies from neighboring districts in the states of Kerala and Maharashtra also reported maximum incidence of cases among adults more than 15 years [11, 12]. This is probably because persons in this age group are more exposed to the causative agent due to the outdoor nature of the work they are engaged in, especially in the forests. The study showed no gender predilection in the reported cases, which is in contrast to the earlier outbreaks in Karnataka where higher proportion of males suffered from KFD probably because they visit the forest resulting in heightened exposure to animals and ticks [4]. In another outbreak in Kerala, more females were found affected with KFD because women accompany the males to the forest for collection of firewood and other forest produce [12]. Similarly, a study in Maharashtra showed higher incidence among females [16]. Thus, we feel that the gender roles in the society determine exposure to the causative agent and hence incidence of KFD.

Second, cases peaked during the winter months of January–April each year. Similar seasonal distribution has also been reported by other studies from India [4, 6, 17]. This is due to the timing of nymphal activity. During the post-monsoon dry season which coincides with the months of January–March, nymphs are more active and are most abundant on the forest floor. Humans get KFD infection mainly through the bite of an infected nymph which explains this seasonal pattern. This period of the year also coincides with cashew harvesting, so people visit forest quite often and are likely to get exposed to the infection. Unlike other diseases, in this study, more than half of the months had no cases reported in a year. Furthermore, the number of cases occurred during specific months also had wide variation. The traditional statistical models may not be effectively applied in this scenario. Hence, to circumvent this challenge, this study used SARIMA as well as ZINB model to forecast KFD incidence, contributing to an early warning system that can help public health policymakers take adequate control measures. Application of ZINB gives an added advantage by simultaneous prediction of time period during which there would be no cases as well as estimating the number of cases that will occur during specific months. We believe that this statistical method has a lot of public health benefits in terms of generating an effective public health response before the actual event/outbreak.

Third, though many case records did not have details related to occupation, available records showed that 94% of cases had a history of travel to forest region. The affected villages are located along the border of the Western Ghats which is one of the important hot spots of bio-diversity in the world. These regions include dense forests and vegetation where people can easily get an exposure to contact with animals and ticks. People go for cashew harvesting during the peak transmission season and often come in contact with ticks and animals such as monkeys. This probably explains the association of travel to forest with the incidence of KFD.

Fourth, the number of cases in each village ranged from 1 per 1000 population to as high as 66 per 1000 population with clustering around Pale, the index village from where cases have been reported initially. Geo-spatial distribution of KFD cases clearly point towards clustering of cases in specific villages (index village) closer to the forest region which could be the focus of public health attention and resources.

Fifth, the case fatality rate in Goa (0.9%) is lesser compared to KFD outbreaks reported from other parts of India (2–10%) [3], the reasons of which cannot be elucidated and could be explored in future studies. But one of the reasons could be that majority of the patients in the present study with the exception of very few were treated at in-patient settings and timely referred to the higher levels, i.e., tertiary hospital, when required.

Sixth, among cases, only 13% had received at least one dose of KFD vaccine which indicates poor vaccination coverage. This might be due to poor implementation of the strategy or lack of community acceptance for the vaccine, the exact reasons requiring a qualitative exploration. A recent mixed method study by Oliveira et al. from the current study area had reported 32% vaccination coverage (for one dose). The reason for poor coverage was found to be inapprpriate timing, poor awareness and various programmatic reasons including vaccine stock [18]. The outbreak from Goa had 3.3% people affected in age group not targeted for vaccination. The study by Kiran et al. from Karnataka also reported 16.9% of KFD cases among the non-targeted group (less than 6 or more than 65 years old) [4]. In light of this evidence, a careful reconsideration of the vaccination strategy is required.

Seventh, the geographical spread of KFD cases shows that the disease spread from 8 villages in 2015 to 31 in 2016 is probably due to lack of immediate public health measures and poor vaccination coverage which started towards the end of 2015 only. Gradually, with the institution of containment measures, health education, and vaccination campaigns, we have been able to restrict the spread to few villages in 2018. However, these efforts need to continue, especially for those who are at high risk of contracting the disease through better vaccination coverage, health education, and other preventive measures.

Eighth, spatial analysis revealed significant spatial auto-correlation in the occurrence of KFD. Local Gi* indicated the spatial extent of hot and cold spots of KFD in the state of Goa. Even though our study did not explore the diffusion pattern of KFD over space and time, it is notable that contiguous pattern of hot and cold spots seemed to predominate. These contiguous hot spots of KFD are the areas for prioritized action and continuous surveillance.

Furthermore, the spatial outliers, low-high or high-low spots, identified in this study could be important regions of future interventions. In contrast to hot spots, these are regions with features having high values that are surrounded by low values or vice versa [15] Therefore, these are pockets where few KFD cases occurred in the midst of villages with more number of KFD cases or vice versa. These spots are likely to diffuse to neighboring areas, warranting closer monitoring and pre-emptive action to prevent the spread.

This is one of the few studies which analyzed more than 400 cases reported in a routine surveillance setting over a period of four consecutive years, thus allowing us to analyze the seasonal trend of occurrence in the state of Goa. Data were double entered and validated which is considered as benchmark to ensure data quality. Another major strength of the study was the robust surveillance system which captured all the laboratory-confirmed cases in the state. This is because there was only one center (MCVR) having testing facility for KFD and any positive result for KFD was routinely reported to the state IDSP unit. Also, all blood samples for testing from both government and private laboratories were mandated to go through the IDSP unit. However, there were few limitations in this study. Firstly, there were missing data related to hospital admission and stay (14.7%), vaccination status (23.4%), and history of travel (68%). Second, since the transmission not only depends on the climate, but also by the occurrence of cases in other animals such as monkeys, rodents, and infective ticks, the forecast considering these features would give robust estimate.

This study has the following programmatic implications. First, since there is clustering of cases around an index village and these villages continue to report cases in subsequent years, there has to be continuous scrutiny of cases in these regions by establishing a comprehensive surveillance system apart from the routine reporting. Second, due to the seasonality of outbreak, we need to draw up an action plan including vaccination strategy that needs to be implemented before the cases start appearing, i.e., before January. This will help in two ways: (i) people will be available for vaccination since that is off season for cashew harvesting and (ii) nymphal activity of ticks will be low before December; thus, people will be in a better immune state when the disease actually starts occurring. Third, the recent increase in the density of cases is a cause for concern. This emphasizes the need to strengthen other preventive measures such as tick control, spray with dimethyl phthalate, and health education to wear long shirts among people who go to the forest region apart from supportive care and vaccination. Implementation of these co-interventions will also benefit the people who come under non-targeted age group for vaccination and people who are not vaccinated. More awareness needs to be spread among the communities living along the forest region, especially those who visit the forest for cashew harvesting (which coincides with the tick transmission season), about the mode of transmission of the disease and preventive measures available. They should also be targeted for full vaccination coverage.

## Conclusion

KFD cases in Goa mostly affected adults more than 15 years of age in both genders and followed a specific seasonal pattern. Clustering of cases occurred in selected villages located in North Goa. Most of the confirmed cases did not receive any vaccination and had history of travel to forest for cashew plucking. Planning for public health interventions such as health education and vaccination campaigns and resource allocation should consider these epidemiological features.

## Supplementary information

**Additional file 1: Table S1.** Forecasted values of number of KFD cases based on Jan 2015-Dec 2018 data for the year 2019.

## Data Availability

The datasets used and analyzed during the current study are available from the corresponding author on request.

## Abbreviations

KFD: Kyasanur Forest Disease
RT-PCR: Reverse transcription-polymerase chain reaction
IDSP: Integrated Disease Surveillance Project
CHC: Community health center
MCVR: Manipal Centre for Virus Research
SARIMA: Seasonal Auto-Regressive Integrated Moving Average model
AR: Auto-regression
MA: Moving average
AIC: Akaike information criteria
