# Supplementary material for: Geospatial clustering, seasonal trend and forecasting of Kyasanur Forest Disease in the state of Goa, India, 2015–2018
Source: Trop Med Health. 2020 Apr 28;48:27. doi: 10.1186/s41182-020-00213-y (PMC7187534; doi:10.1186/s41182-020-00213-y)
Supplement: Supplementary file 1 — Additional file 1: Table S1. Forecasted values of number of KFD cases based on Jan 2015-Dec 2018 data for the year 2019. [file 41182_2020_213_MOESM1_ESM.docx]

Supplementary Table 1: Forecasted values of number of KFD cases based on Jan 2015-Dec 2018 data for the year 2019

| Month | Predicted values | Lower limit | Upper limit |
| --- | --- | --- | --- |
| 2019-Jan | 13.9 | 0 | 31.3 |
| 2019-Feb | 6 | 0 | 24.1 |
| 2019-Mar | 14.4 | 0 | 37.9 |
| 2019-Apr | 21.6 | 0 | 45.4 |
| 2019-May | 4 | 0 | 18.1 |
| 2019-Jun | 0 | 0 | 0 |
| 2019-July | 0 | 0 | 0 |
| 2019-Aug | 0 | 0 | 0 |
| 2019-Sep | 0 | 0 | 0 |
| 2019-Oct | 0 | 0 | 0 |
| 2019-Nov | 0 | 0 | 0 |
| 2019-Dec | 0 | 0 | 0 |
